# The influence of physical activity self-efficacy on the continuance use of AI learning tools among physical education students: a three-wave longitudinal study

**DOI:** 10.3389/fpsyg.2026.1844939

**Published:** 2026-07-23

**Authors:** Junhao Zhang, Lu Zhen, Junhui Zhu, Mujuan Zhao, Jing Li, Fang Tan

**Affiliations:** 1 Department of Physical Education, Guizhou Medical University, Guiyang, China; 2 Department of Physical Training and Sports, Saint Euphrosyne Polotsk State University, Polotsk, Belarus; 3 Department of Physical Education, Faculty of Education, Universiti Kebangsaan Malaysia, Bangi, Malaysia; 4 Department of Physical Education, Kyungpook National University, Sangju, Republic of Korea; 5 Department of Physical Education, Sichuan Tianfu New District Experimental Primary School, Chengdu, China; 6 Department of Physical Education, State University of Jakarta, Jakarta, Indonesia; 7 Department of Medicine and Research, Lijiang College of Culture and Tourism, Lijiang, China

**Keywords:** AI learning tools, Continuance use behavior, Physical activity self-efficacy, Physical education students, Technology acceptance model

## Abstract

**Objective:**

Grounded in the context of physical education and based on the Technology Acceptance Model (TAM), this study examined the time-separated associations between physical activity self-efficacy and the continuance use of AI learning tools among physical education students through multiple chained mediation pathways involving perceived ease of use, perceived usefulness, attitude toward use, and behavioral intention to use.

**Methods:**

A three-wave longitudinal design was employed. Data were collected from physical education students in April 2025 (T1), July 2025 (T2), and November 2025 (T3), resulting in 511 successfully matched samples. Physical activity self-efficacy was measured at T1; perceived ease of use, perceived usefulness, attitude toward use, and behavioral intention to use were measured at T2; and continuance use behavior was measured at T3. Structural equation modeling (SEM) and the Bootstrap method (5,000 resamples) were used to test multiple chained mediation effects within the TAM framework.

**Results:**

All five chained mediation pathways (H1–H5) were statistically significant, and the overall chained mediation effect was 0.113 (95% CI [0.083, 0.143]). The chained mediation effect for path aegh was significant (β = 0.011, 95% CI [0.006, 0.018], *p* < 0.01). The serial mediation effect for path afh was significant (β = 0.047, 95% CI [0.029, 0.067], *p* < 0.01). The chained mediation effect for path bdgh was significant (β = 0.022, 95% CI [0.013, 0.034], *p* < 0.01). The chained mediation effect for path bcegh was significant (β = 0.006, 95% CI [0.004, 0.011], *p* < 0.001). The chained mediation effect for path bcfh was also significant (β = 0.027, 95% CI [0.018, 0.040], *p* < 0.01).

**Conclusion:**

Among Chinese physical education students, the continuance use of AI learning tools appears to be associated with physical activity self-efficacy through a time-separated process involving perceived ease of use, perceived usefulness, attitude toward use, and behavioral intention to use. Based on three-wave longitudinal data, this study provides evidence that physical activity self-efficacy may function as a domain-specific psychological antecedent associated with self-reported continuance use behavior through TAM-based cognitive, attitudinal, and intentional processes in the context of physical education.

## 1 Background

### 1.1 Rise of AI learning tools in educational contexts

In recent years, AI tools designed for learning scenarios have rapidly proliferated, with applications covering intelligent question answering, learning planning, assignment feedback, content recommendation, learning analytics, and, in some cases, training-related feedback or learning support (Luo et al., 2025). In this study, AI learning tools refer to AI-supported digital or hardware-based tools that students use to assist learning, feedback seeking, task completion, and training-related planning or reflection in physical education contexts; they are not assumed to provide professional physiological monitoring or real-time biomechanical coaching in all cases. As these tools have been increasingly integrated into educational practice, students have begun to use them as supportive resources in daily learning. One major advantage of AI tools lies in their ability to provide immediate support during the learning process, partially transforming prompts, explanations, and feedback that traditionally depended on teachers or peers into digital services that are available at any time (Merino-Campos, 2025).

However, educational practice has shown that the availability of a tool does not automatically translate into ease of use, nor does ease of use necessarily guarantee continued use. Although many tools may initially attract students, their use may decline as course difficulty increases, learning tasks become more complex, and user experiences fluctuate (Han et al., 2024). Therefore, whether AI learning tools can realize their educational potential depends largely on students’ stable continuance use behavior (Sabani et al., 2025). Understanding such behavior requires attention not only to tool characteristics, but also to the psychological mechanisms underlying students’ cognition, attitudes, and behavioral intentions.

### 1.2 Unique characteristics of physical education students

The learning tasks of physical education students are characterized by a strong practice-oriented nature, in which motor skills, physical training, tactical understanding, classroom practice, and course assessment are often closely intertwined. Unlike learning that primarily emphasizes knowledge memorization, physical education places greater emphasis on learning through practice, a process that is highly dependent on feedback. Whether students can obtain actionable feedback after practice often affects the effectiveness of subsequent training (Su et al., 2025).

In this context, AI learning tools should not be understood as replacements for coaches or physiological monitoring systems, but as supplementary resources that may support information access, feedback seeking, exercise planning, reflective learning, and task completion. Therefore, physical education students constitute a relevant population for examining the continuance use behavior of AI learning tools, particularly in relation to students’ perceived usefulness, perceived ease of use, and willingness to use such tools as supplementary learning resources (Soliman et al., 2025; Chen et al., 2026).

In addition, physical education students’ learning and training often show stage-based and cyclical patterns, such as semester course arrangements, specialized training processes, and competition preparation cycles. These characteristics make this group suitable for observing the transition from initial trial to sustained use of AI learning tools (Yaseen et al., 2025).

### 1.3 Role of physical activity self-efficacy in TAM

Physical activity self-efficacy refers to individuals’ belief in their ability to maintain participation in physical activity, complete exercise-related tasks, and overcome relevant barriers (Bandura, 1997). In the context of physical education, this belief is related not only to students’ persistence in training and course completion, but also to their acceptance of emerging learning tools (Huang et al., 2025; Sheng et al., 2025). Compared with general learners, physical education students’ learning tasks place greater emphasis on motor practice, physical development, specialized skill mastery, and feedback-based improvement (Gao et al., 2025). Students with higher self-efficacy are generally more willing to actively try new supportive resources and, when encountering difficulties, are more likely to solve problems by adjusting strategies and sustaining practice rather than giving up easily (Bateman et al., 2022). For this reason, physical activity self-efficacy is not only an important psychological variable for explaining persistence in physical activity, but may also serve as a domain-specific antecedent for understanding whether physical education students are willing to continue using AI learning tools. This is because, in physical education, AI learning tools may be used to support feedback seeking, learning reflection, exercise planning, knowledge acquisition, and task completion, which are closely connected with students’ physical activity and training experiences, although such support does not necessarily constitute individualized professional training prescription (Ren et al., 2025; Wang and Chuang, 2024).

From the perspective of the Technology Acceptance Model (TAM), physical activity self-efficacy may further influence the process of continuance adoption by shaping learners’ cognitive evaluations of AI tools. On the one hand, students with higher physical activity self-efficacy are more likely to believe that they can cope with learning and training challenges, actively seek feedback, and adjust their learning strategies. These tendencies may make them more likely to perceive AI learning tools as manageable and controllable, thereby developing higher perceived ease of use. On the other hand, because students with higher physical activity self-efficacy tend to place greater value on resources that can improve training quality and learning efficiency, they may be more likely to regard AI tools as potentially useful supplementary resources for supporting training-related reflection, knowledge acquisition, and task completion, thereby developing higher perceived usefulness. As these positive cognitive evaluations accumulate, learners are likely to develop more favorable attitudes toward use, further strengthen their behavioral intention to use, and ultimately translate these intentions into stable continuance use behavior (Ni and Cheung, 2023; Jeong et al., 2025). In other words, this study does not assume that physical activity self-efficacy directly represents technology-related ability. Instead, it conceptualizes physical activity self-efficacy as a domain-specific psychological resource that may be linked to technology-related behavior through feedback seeking, perceived tool control, and evaluations of perceived learning value, without assuming that AI tools can independently provide scientifically validated or fully personalized training prescriptions.

### 1.4 Limitations of previous research

Existing studies on the adoption of AI learning tools have provided an important foundation, but several theoretical and methodological gaps remain unresolved. First, many studies have adopted cross-sectional designs, which are useful for describing correlations but limited in clarifying temporal order and dynamic mechanisms. This limitation is not merely methodological; it also restricts theoretical understanding of how students move from initial cognitive evaluation to sustained use over time (Podsakoff et al., 2003; Ployhart and Vandenberg, 2010).

Second, many studies have treated usage intention as the primary outcome variable, while relatively few have incorporated continuance use behavior into the model. Intention is important, but in real learning settings, a gap often exists between intention and behavior, and sustained use may still depend on specific learning experiences and contextual constraints (Sheeran, 2002; Webb and Sheeran, 2006). Although continuance use has often been examined through expectation-confirmation models, the present study focuses on the pre-use and ongoing acceptance process emphasized by TAM, particularly how domain-specific self-efficacy shapes perceived ease of use, perceived usefulness, attitude, and intention before being translated into self-reported continuance use behavior.

Third, in terms of sample selection, some studies have relied on broad student populations or general adult users, which may lead to overgeneralized explanations of the underlying mechanisms. Such samples make it difficult to determine whether self-efficacy operates as a general individual trait or as a context-specific antecedent in skill-learning contexts that are highly dependent on feedback. Therefore, in a physical education context that is more closely tied to training and practice, it is clearly necessary to adopt a longitudinal design that can capture temporal order and to include continuance use behavior as an outcome variable when examining the mechanisms underlying the sustained adoption of AI learning tools (Maxwell and Cole, 2007). More importantly, existing TAM-based studies have rarely explained how a physical-domain efficacy belief can be translated into technology-related continuance behavior, leaving unclear whether self-efficacy functions merely as a general individual trait or as a context-specific psychological antecedent in AI-supported physical education learning.

### 1.5 Research model and hypotheses

**FIGURE 1:**
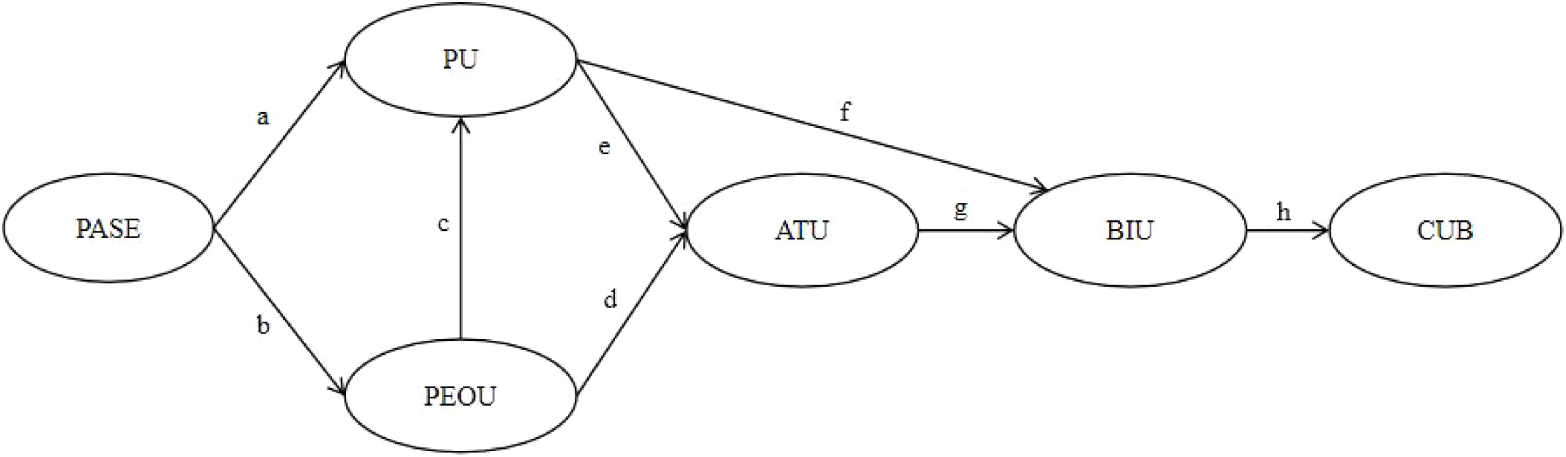
Proposed research model. PASE, Physical activity self-efficacy; PU, perceived usefulness; PEOU, perceived ease of use; ATU, attitude toward use; BIU, behavioral intention to use; CUB, continuance use behavior.

This study focused on physical education students and examined how physical activity self-efficacy, as a domain-specific belief rooted in training and physical activity experiences, is associated with continuance use behavior of AI learning tools through the TAM-based chain of perceived ease of use, perceived usefulness, attitude toward use, and behavioral intention to use. In this model, physical activity self-efficacy is not treated as a direct indicator of technology competence, but as an action-oriented belief that may influence students’ perceptions of tool controllability and learning value in AI-supported physical education. Although the hypothesized paths follow the established sequential logic of TAM, the purpose of this study was not to propose a completely new acceptance sequence, but to examine whether this sequence can explain the cross-domain transformation from physical activity self-efficacy to AI learning tool continuance use in a longitudinal physical education context. As shown below, research hypotheses H1–H5 were proposed, and the research model was constructed (Figure 1), with the model paths coded from a to h.

H1: Physical activity self-efficacy positively influences continuance use behavior through the chain mediation of perceived usefulness, attitude toward use, and behavioral intention to use.

H2: Physical activity self-efficacy positively influences continuance use behavior through the mediation of perceived usefulness and behavioral intention to use.

H3: Physical activity self-efficacy positively influences continuance use behavior through the chain mediation of perceived ease of use, attitude toward use, and behavioral intention to use.

H4: Physical activity self-efficacy positively influences continuance use behavior through the chain mediation of perceived ease of use, perceived usefulness, attitude toward use, and behavioral intention to use.

H5: Physical activity self-efficacy positively influences continuance use behavior through the chain mediation of perceived ease of use, perceived usefulness, and behavioral intention to use.

## 2 Methods

### 2.1 Participants

A combination of convenience sampling and snowball sampling was used, with full-time physical education students in China serving as the target population. This sampling strategy was adopted because the study required the same participants to complete three waves of questionnaires over several months, and direct access to a complete national sampling frame of physical education students was not available. Questionnaires were distributed both online (via Wenjuanxing and WeChat) and offline (on university campuses), covering participants with diverse backgrounds in terms of age, academic level, city category, and use of AI learning tools, in order to enhance the informational diversity of the sample (Table 1). To improve the authenticity and completeness of responses, the survey was conducted anonymously. However, because this was a non-probability sampling strategy, potential selection bias cannot be completely ruled out.

**TABLE 1:** Sample characteristics.

Variable	Category	Frequency%	Variable	Category	Frequency%
Gender	Male	297 (58.1)	Academic level	Undergraduate	255 (49.9)
	Female	214 (41.9)		Graduate student	256 (50.1)
Age	18 years	75 (14.7)	City category	Developed cities	272 (53.2)
	19 years	71 (13.9)		Less-developed cities	239 (46.8)
	20 years	55 (10.7)	Frequent use of AI learning tools	Yes	454 (88.8)
	21 years	80 (15.7)		No	57 (11.2)
	22 years	47 (9.2)	Type of AI tools used most frequently	Hardware-based AI tools	200 (39.1)
	23 years and above	183 (35.8)		Software-based AI tools	311 (60.9)

#### 2.1.1 Sample size estimation

Structural equation modeling (SEM) was used for hypothesis testing. According to the rule of thumb, the sample size required for SEM is typically estimated based on the number of measurement items, with approximately 10 samples needed for each observed item (Hair et al., 2019). The questionnaire included 47 items, resulting in a minimum required sample size of 470 (47 × 10 = 470) to ensure the stability of model fit and path estimation. Considering that the model also involved a chained mediation structure, the recommendation by Kline (2023) of a minimum sample size of 500 was further taken into account. Therefore, the target sample size was set at 500. In the present study, 511 samples were successfully matched across the three waves (> 500), indicating that the sample size was sufficient to meet the requirements for data analysis.

#### 2.1.2 Sample characteristics

All 511 participants were physical education students aged 18 years or older. Among them, 297 were male (58.1%) and 214 were female (41.9%). The remaining sample characteristics are presented in Table 1. In terms of AI learning tools, software-based tools mainly included AI question-answering systems, learning-planning applications, assignment-feedback tools, and content-recommendation systems, whereas hardware-based tools mainly referred to AI-supported wearable devices, smart training equipment, and motion-feedback devices used in learning or training contexts.

### 2.2 Research design

A three-wave longitudinal tracking design was adopted, in which the same group of participants was measured in April 2025 (T1), July 2025 (T2), and November 2025 (T3), with a three-month interval between each wave. To enable cross-wave matching, a unique anonymous identification code was generated for each participant at T1 and used for subsequent matching. The three-wave design made it possible to examine the temporal ordering of the hypothesized variables and to test time-separated mediation pathways. Compared with cross-sectional studies, this design is more conducive to identifying temporal ordering among variables and may help reduce, although not eliminate, the influence of common method bias and reverse causality (Maxwell et al., 2011; Selig and Preacher, 2009). In addition, prior empirical studies have also employed three-wave time-lagged designs to verify the mechanisms underlying mediation pathways, thereby providing a direct methodological reference for the present study (Haider et al., 2019).

At T1, the independent variable, physical activity self-efficacy, was measured. At T2, the mediating variables, namely perceived ease of use, perceived usefulness, attitude toward use, and behavioral intention to use, were assessed. At T3, the dependent variable, continuance use behavior, was measured. This design followed the temporal sequence of “independent variable–mediator–dependent variable,” which helped clarify the temporal direction of the hypothesized relationships and reduce potential biases resulting from synchronous measurement (Maxwell and Cole, 2007; Podsakoff et al., 2003). Through this design, the identification of model pathways was ensured, while also avoiding overparameterization problems caused by limitations in sample size and the number of measurement items (Little, 2013).

A total of 545 responses were collected at Wave 1 (T1), 526 at Wave 2 (T2), and 511 at Wave 3 (T3), yielding a retention rate of 96.5% from T1 to T2 and 97.2% from T2 to T3. The overall three-wave panel retention rate was 93.8% (511/545). Sample attrition was mainly due to changes in contact information, failure to complete the questionnaire on time, and voluntary withdrawal. Ultimately, the 511 successfully matched samples were used for reliability, validity, and structural equation model analyses.

### 2.3 Measures

Six scales were used in this study. The questionnaire was adapted from well-established theories and previously validated instruments, with localized semantic modifications made for the context of AI learning tools and physical education students. All scales were specified as first-order unidimensional constructs and were measured using a 7-point Likert scale (1 = strongly disagree, 7 = strongly agree). To improve content validity and contextual appropriateness, the item pool was reviewed by researchers in physical education and educational technology, pretested with physical education students, and checked by bilingual researchers for conceptual equivalence and cultural suitability.

*Physical activity self-efficacy.* This construct was measured with 9 items. Drawing on Bandura’s (1997) self-efficacy theory and the measurement framework for exercise self-efficacy, the scale was adapted to fit the learning and training context of physical education students. It was used to assess their beliefs in their ability to persist in physical activity, complete training tasks, and overcome related barriers.

*Perceived ease of use and perceived usefulness.* Based on the Technology Acceptance Model (Davis, 1989), a perceived ease of use scale (7 items) and a perceived usefulness scale (9 items) were developed. Perceived ease of use was used to measure learners’ subjective experiences regarding the simplicity of operation, interface friendliness, and smoothness of interaction when using AI learning tools. Perceived usefulness was used to assess learners’ judgments of the performance-enhancing value of AI learning tools in completing learning or training tasks. Together, these two constructs reflect the core cognitive dimensions of learners’ acceptance of AI learning tools.

*Attitude toward use and behavioral intention to use.* Based on the mediating roles of attitude and intention in the technology adoption process within TAM (Davis et al., 1989; Venkatesh and Davis, 2000), an attitude toward use scale (6 items) and a behavioral intention to use scale (8 items) were developed. The attitude toward use scale was used to assess learners’ affective evaluations of AI learning tools. The behavioral intention to use scale measured learners’ willingness to continue using AI tools in the future. Together, these two scales reflect learners’ affective cognition and behavioral intention and serve as key mediating variables in the TAM framework, linking perceived ease of use and perceived usefulness to continuance use behavior.

Continuance use behavior. Based on the Unified Theory of Acceptance and Use of Technology (Venkatesh et al., 2003) and its extended model (Venkatesh et al., 2012), continuance use behavior was measured using an 8-item self-report scale, focusing primarily on learners’ perceived frequency, duration, and functional scope of use during the learning process. Because objective platform logs were not available in the present study, this variable should be interpreted as self-reported continuance use behavior rather than objectively recorded usage behavior.

Although a large sample size and numerous measurement items may inflate the χ^2^ statistic and affect model fit, Table 2 indicates that both the measurement instruments and the overall model demonstrated acceptable fit and reliability.

**TABLE 2:** Model fit indices and reliability analysis.

Variable/Model	χ*^2^*	*df*	χ*^2^*/df	GFI	AGFI	NFI	RMSEA	Cronbach’s α
PASE	22.507	27	0.834	0.990	0.984	0.993	0.010	0.928
PEOU	19.198	14	1.371	0.990	0.979	0.992	0.027	0.915
PU	32.837	27	1.216	0.986	0.977	0.989	0.021	0.927
ATU	12.224	9	1.358	0.992	0.982	0.993	0.027	0.898
BIU	23.493	20	1.175	0.988	0.979	0.992	0.017	0.933
CUB	27.767	20	1.388	0.987	0.977	0.991	0.017	0.929
Overall Model	1346.967	1026	1.313	0.906	0.896	0.927	0.025	–

Model evaluation criteria: χ^2^/df ≤ 3; GFI ≥ 0.90; AGFI close to or above 0.90; NFI ≥ 0.90; RMSEA ≤ 0.08; Cronbach’s α ≥ 0.70.

### 2.4 Data analysis

To ensure the reliability and validity of the measurement model, multiple statistical methods were employed to systematically test the chained mediation effects based on the Technology Acceptance Model (TAM), with the significance level set at *p* < 0.05.

First, to examine the potential presence of common method bias (CMB), AMOS 26.0 was used to construct a single-factor confirmatory factor analysis model, in which all measurement items were loaded onto one latent factor. This test was used as a diagnostic procedure rather than as definitive evidence that shared method variance was fully absent.

Second, exploratory factor analysis (EFA) and confirmatory factor analysis (CFA) were conducted on split-half samples to examine the factor structure of the six latent variables and the structural validity of the measurement model. Items with standardized factor loadings greater than 0.50 were retained. The results showed that all models demonstrated good fit.

Subsequently, the reliability and validity of the model were further assessed by jointly examining the CFA results, composite reliability (CR), average variance extracted (AVE), and the square roots of the AVE values. In addition, IBM SPSS 26.0 was used to perform correlation analysis and descriptive statistical analysis in order to examine the preliminary associations among the latent variables and their distributional characteristics.

On this basis, to test the chained mediation effects, a structural equation model (SEM) was constructed in AMOS 26.0, and the model paths were uniformly coded from a to h. The chained mediation pathways were specified through AmosEstimandVB backend programming, and the Bootstrap method (5,000 resamples) was used to estimate bias-corrected 95% confidence intervals for the path coefficients, thereby testing the significance of the chained mediation effects proposed in Hypotheses H1–H5. Finally, Microsoft PowerPoint 2019 was used to draw the model diagram containing standardized path coefficients in order to enhance the clarity and readability of the results presentation.

### 2.5 Research procedure

**FIGURE 2:**
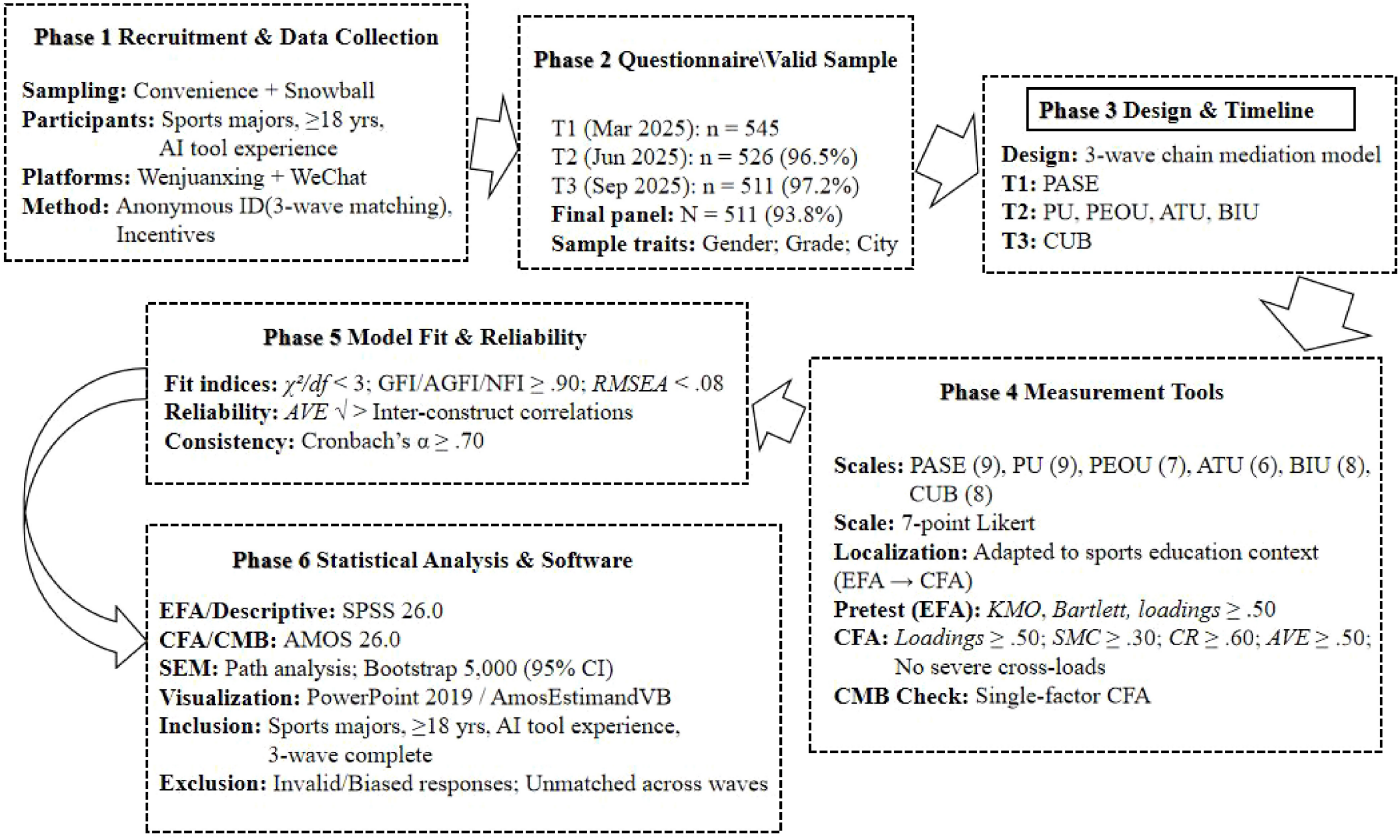
Research process.

The overall research procedure was divided into six phases (Figure 2). Phases 1–2 involved the recruitment of physical education students, three-wave matching, and the screening of valid samples. Phases 3–4 focused on the construction of a TAM-based chained mediation model with physical activity self-efficacy as an external variable and the implementation of scale validation. Phases 5–6 evaluated model quality based on fit indices as well as reliability and validity indicators, and tested the stability of each pathway through structural equation modeling and Bootstrap analyses.

## 3 Results

### 3.1 Common method bias

To examine whether common method bias was present, a single-factor confirmatory factor analysis was conducted using AMOS (Podsakoff et al., 2003). All measurement variables were loaded onto one latent factor to construct a single-factor model. The results showed poor model fit: χ*^2^* = 9972.236, *df* = 1034, χ*^2^/df* = 9.644, GFI = 0.387, AGFI = 0.331, NFI = 0.458, and RMSEA = 0.130, none of which met the model fit criteria proposed by Hu and Bentler (1999) (χ*^2^/df* < 3, GFI, AGFI, and NFI ≥ 0.90, RMSEA ≤ 0.08). These findings indicate that a single factor could not adequately account for the variance among all measurement variables. Subsequently, a complete measurement model including all latent variables was tested, and the results showed good model fit: χ*^2^* = 1346.967, *df* = 1026, χ*^2^/df* = 1.313, GFI = 0.906, AGFI = 0.896, NFI = 0.927, and RMSEA = 0.025. Therefore, the single-factor CFA results did not indicate serious common method bias in this study. However, because all variables were measured using self-report questionnaires, the possibility of shared method variance cannot be completely ruled out.

### 3.2 Exploratory factor analysis

The Kaiser–Meyer–Olkin (KMO) value was 0.955, and Bartlett’s test of sphericity was significant, χ*^2^*(1,081) = 17,809.230, *p* < 0.001. Factor extraction was conducted using split-half samples. Based on the criterion of eigenvalues greater than 1 and the scree plot results, six common factors were ultimately extracted. The initial eigenvalues of the six factors were 16.538, 3.643, 3.229, 2.831, 2.743, and 2.169, respectively, with a cumulative variance contribution rate of 66.286%, indicating that the extracted factor structure had strong explanatory power. After rotation, the variance contributions of the six factors were relatively balanced.

The item loadings of each latent variable on their respective factors were all relatively high. Specifically, the factor loadings ranged from 0.704 to 0.862 for physical activity self-efficacy, 0.707 to 0.860 for perceived usefulness, 0.713 to 0.843 for perceived ease of use, 0.696 to 0.826 for attitude toward use, 0.713 to 0.853 for behavioral intention to use, and 0.691 to 0.854 for continuance use behavior. Overall, the loading structure of the six factors was clear and consistent with the theoretical model, indicating that the scales demonstrated good structural validity.

### 3.3 Confirmatory factor analysis

Confirmatory factor analysis was conducted for all observed indicators. Following the recommendation of Miao and Zhang (2024), observed items with factor loadings greater than 0.50 were retained. After screening, the CFA, SMC, CR, and AVE results are presented in Table 3. In this study, the standardized factor loadings (Std.) of all items ranged from 0.715 to 0.984, indicating significant associations between the observed variables and their corresponding latent variables. The composite reliability (CR) values of the latent variables ranged from 0.900 to 0.934, suggesting strong internal consistency and good correlations among the indicators of each construct. The average variance extracted (AVE) values ranged from 0.593 to 0.641, indicating that the latent variables explained a substantial proportion of the variance in their observed indicators and thus demonstrated satisfactory convergent validity overall. In addition, Cronbach’s α coefficients for all scales ranged from 0.898 to 0.933, further supporting the high internal consistency of the measures.

**TABLE 3:** Confirmatory factor analysis results and psychometric properties.

Item	UnStd.	S.E.	C.R.	*p*	Std.	SMC	CR	AVE	Cronbach α
T1_PASE1	1				0.970	0.941	0.930	0.597	0.928
T1_PASE2	0.829	0.033	24.460	***	0.759	0.576			
T1_PASE3	0.762	0.033	22.705	***	0.732	0.535			
T1_PASE4	0.809	0.033	23.957	***	0.751	0.565			
T1_PASE5	0.787	0.033	23.670	***	0.747	0.558			
T1_PASE6	0.797	0.033	23.723	***	0.748	0.559			
T1_PASE7	0.746	0.033	22.336	***	0.725	0.526			
T1_PASE8	0.789	0.033	23.428	***	0.743	0.553			
T1_PASE9	0.808	0.034	23.318	***	0.741	0.550			
T2_PEOU1	1				0.970	0.942	0.917	0.614	0.915
T2_PEOU2	0.748	0.033	22.628	***	0.734	0.539			
T2_PEOU3	0.740	0.031	23.317	***	0.746	0.556			
T2_PEOU4	0.797	0.033	24.073	***	0.758	0.574			
T2_PEOU5	0.772	0.033	22.968	***	0.740	0.548			
T2_PEOU6	0.744	0.032	22.979	***	0.740	0.548			
T2_PEOU7	0.797	0.032	24.580	***	0.765	0.586			
T2_PU1	1				0.975	0.950	0.928	0.593	0.927
T2_PU2	0.755	0.032	23.116	***	0.736	0.541			
T2_PU3	0.814	0.033	23.967	***	0.749	0.561			
T2_PU4	0.731	0.032	22.399	***	0.724	0.524			
T2_PU5	0.733	0.033	21.861	***	0.715	0.511			
T2_PU6	0.764	0.033	22.687	***	0.729	0.531			
T2_PU7	0.761	0.032	23.660	***	0.744	0.554			
T2_PU8	0.813	0.033	24.413	***	0.755	0.571			
T2_PU9	0.790	0.031	25.081	***	0.765	0.586			
T2_ATU1	1				0.984	0.969	0.900	0.604	0.898
T2_ATU2	0.736	0.032	22.318	***	0.727	0.529			
T2_ATU3	0.721	0.032	22.171	***	0.724	0.525			
T2_ATU4	0.743	0.031	23.263	***	0.743	0.552			
T2_ATU5	0.710	0.032	21.816	***	0.718	0.516			
T2_ATU6	0.711	0.032	22.242	***	0.726	0.527			
T2_BIU1	1				0.980	0.961	0.934	0.641	0.933
T2_BIU2	0.809	0.030	26.235	***	0.776	0.603			
T2_BIU3	0.829	0.031	26.744	***	0.783	0.613			
T2_BIU4	0.791	0.032	24.674	***	0.755	0.571			
T2_BIU5	0.815	0.030	26.638	***	0.781	0.611			
T2_BIU6	0.776	0.031	24.284	***	0.750	0.562			
T2_BIU7	0.829	0.031	26.235	***	0.776	0.603			
T2_BIU8	0.812	0.030	26.289	***	0.777	0.604			
T3_CUB1	1				0.977	0.956	0.930	0.626	0.929
T3_CUB2	1.018	0.053	18.907	***	0.764	0.584			
T3_CUB3	1.022	0.054	18.635	***	0.755	0.570			
T3_CUB4	1.022	0.053	19.103	***	0.770	0.594			
T3_CUB5	0.973	0.053	18.326	***	0.745	0.555			
T3_CUB6	0.971	0.052	18.681	***	0.756	0.573			
T3_CUB7	0.979	0.052	18.631	***	0.755	0.570			
T3_CUB8	1.272	0.049	25.858	***	0.774	0.599			

***p < 0.001.

The six latent variables—physical activity self-efficacy, perceived usefulness, perceived ease of use, attitude toward use, behavioral intention to use, and self-reported continuance use behavior—demonstrated high levels of reliability and convergent validity, meeting the criteria proposed by Fornell and Larcker (1981), namely standardized factor loadings (Std.) > 0.50, CR > 0.60, and AVE ≥ 0.50. These results provide empirical support for the construct validity of the adapted questionnaire in the present research context.

### 3.4 Correlation analysis and descriptive statistics

As shown in Table 4, the mean scores (M) of the six latent variables ranged from 3.788 to 4.426, indicating that the variables, from physical activity self-efficacy to continuance use behavior, were generally at a moderate to moderately high level. The absolute values of skewness ranged from 0.069 to 0.195, and the absolute values of kurtosis ranged from 1.315 to 1.428. These values did not exceed the commonly accepted thresholds of an absolute skewness value below 2 and an absolute kurtosis value below 8, suggesting that the data approximately satisfied the assumption of normal distribution.

**TABLE 4:** Correlations and descriptive statistics.

Variable	1	2	3	4	5	6	M	SD	Skewness	Kurtosis	AVE
1. PASE	***0.773***	***0.784***	***0.770***	***0.777***	***0.801***	***0.791***	3.788	1.466	0.170	−1.321	0.597
2. PEOU	0.389**						4.272	1.526	−0.069	−1.315	0.614
3. PU	0.392**	0.454**					3.695	1.477	0.195	−1.322	0.593
4. ATU	0.454**	0.451**	0.393**				4.326	1.598	−0.013	−1.332	0.604
5. BIU	0.455**	0.369**	0.479**	0.441**			4.426	1.701	−0.108	−1.428	0.641
6. CUB	0.488**	0.413**	0.436**	0.404**	0.490**		4.314	1.501	−0.092	−1.336	0.626

**p < 0.01; Values on the diagonal are the square roots of AVE.

In addition, discriminant validity among the six variables was further examined using AVE. The square root of the AVE for each variable was greater than its standardized correlation coefficients with the other variables (shown in bold on the diagonal), indicating that the latent variables involved in this study demonstrated good discriminant validity.

### 3.5 Chained mediation analysis

**FIGURE 3:**
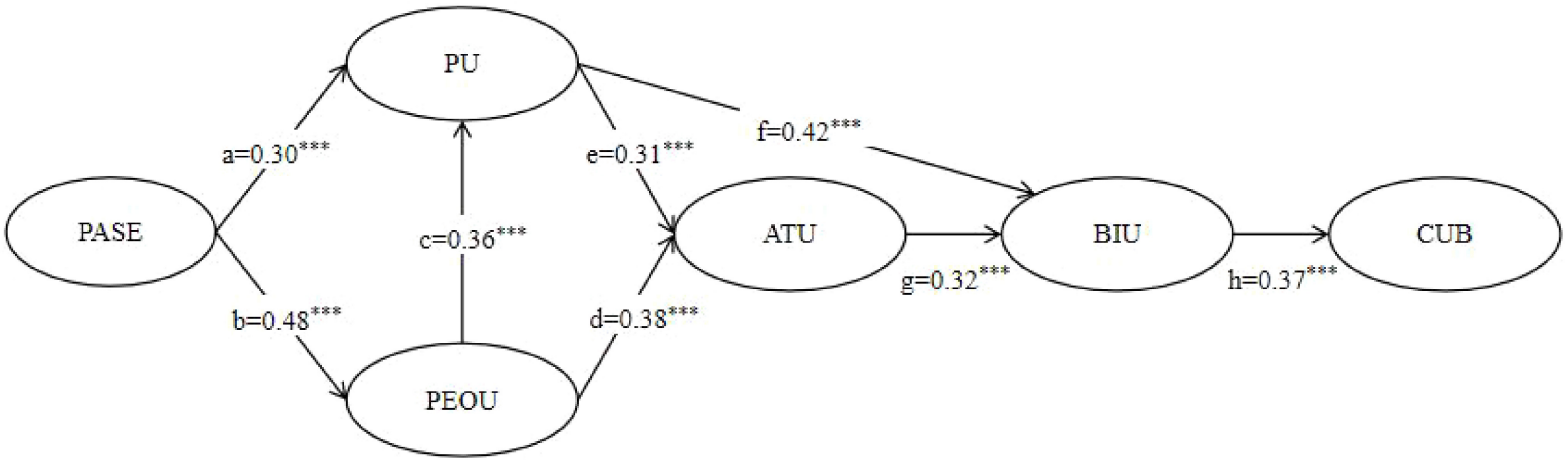
Chain mediation pathway diagram under the TAM framework. ***p < 0.001; T1: PASE; T2: PEOU, PU, ATU, and BIU; T3: CUB.

As shown in Figure 3, the path results indicated that physical activity self-efficacy significantly and positively predicted perceived usefulness, with path coefficient a (γ = 0.30, *p* < 0.001), and perceived ease of use, with path coefficient b (γ = 0.48, *p* < 0.001), suggesting that physical activity self-efficacy significantly and positively influenced individuals’ perceptions of AI learning tools. The path coefficient from perceived ease of use to perceived usefulness, c, was γ = 0.36 (*p* < 0.001); the path coefficient from perceived ease of use to attitude toward use, d, was γ = 0.38 (*p* < 0.001); and the path coefficient from perceived usefulness to attitude toward use, e, was γ = 0.31 (*p* < 0.001). These results suggest that perceived ease of use of AI learning tools was positively associated with perceived usefulness, and that both perceived usefulness and perceived ease of use were positively associated with individuals’ attitudes toward AI learning tools.

The path coefficient from perceived usefulness to behavioral intention to use, f, was γ = 0.42 (*p* < 0.001), indicating a direct and positive effect of perceived usefulness on behavioral intention to use. The path coefficient from attitude toward use to behavioral intention to use, g, was γ = 0.32 (*p* < 0.001), and the path coefficient from behavioral intention to use to continuance use behavior, h, was γ = 0.37 (*p* < 0.001), suggesting that attitude and intention were significantly and positively associated with subsequent self-reported continuance use behavior. All of the above direct effects were positive and significant, thereby providing a basis for further testing of the chained mediation effects.

The Bootstrap test results shown in Table 5 (5,000 resamples) indicated that the overall chained mediation effect was significant (β = 0.113, 95% CI [0.083, 0.143], *p* < 0.01, *Z* > 1.96). The chained mediation effect for path aegh was significant (β = 0.011, 95% CI [0.006, 0.018], *p* < 0.01). The serial mediation effect for path afh was significant (β = 0.047, 95% CI [0.029, 0.067], *p* < 0.01). The chained mediation effect for path bdgh was significant (β = 0.022, 95% CI [0.013, 0.034], *p* < 0.01). The chained effect for path bcegh was significant (β = 0.006, 95% CI [0.004, 0.011], *p* < 0.001). The chained effect for path bcfh was also significant (β = 0.027, 95% CI [0.018, 0.040], *p* < 0.01).

**TABLE 5:** Longitudinal chain mediation effects under the TAM framework.

Hypothesis	Indirect path	β	Product of coefficients		Bias-corrected	
					95% CI	
			*S.E*	*Z*	Lower	Upper
H1	a→e→g→h	0.011**	0.003	3.422	0.006	0.018
H2	a→f→h	0.047**	0.010	4.795	0.029	0.067
H3	b→d→g→h	0.022**	0.005	4.028	0.013	0.034
H4	b→c→e→g→h	0.006***	0.002	4.083	0.004	0.011
H5	b→c→f→h	0.027**	0.006	4.920	0.018	0.040
Total		0.113**	0.015	7.695	0.083	0.143

**p < 0.01; ***p < 0.001.

Overall, the mediation effects were statistically significant but modest in magnitude. This suggests that physical activity self-efficacy may be associated with the continuance use behavior of AI learning tools through a stable but relatively limited chained mediation pathway. Therefore, H1–H5 were supported; however, these results should not be interpreted solely based on statistical significance. Greater emphasis should be placed on the modest effect sizes, the sequential psychological process, and a cautious interpretation of the practical implications, particularly when translating these findings into educational practice.

## 4 Discussion

### 4.1 Overview of the main findings

Based on a three-wave longitudinal design, this study systematically examined the time-separated mediation pathways through which physical activity self-efficacy is associated with the continuance use behavior of AI learning tools among physical education students. The results showed that physical activity self-efficacy not only significantly and positively predicted perceived ease of use and perceived usefulness, but was also associated with self-reported continuance use behavior through multiple chained mediation pathways involving perceived ease of use, perceived usefulness, attitude toward use, and behavioral intention to use. These findings suggest that physical activity self-efficacy is not merely a general individual-difference variable, but a context-specific action belief that may shape how physical education students evaluate and engage with AI learning tools.

This finding does more than support the applicability of TAM in physical education settings. It suggests that technology acceptance in AI-supported physical education should be understood as a process shaped by both tool-related evaluations and learners’ preexisting action beliefs. Among physical education students, who are characterized by strong practical demands, reliance on feedback, and challenging training tasks, physical activity self-efficacy may enter the TAM process by influencing perceptions of tool controllability and learning value. This implies that continuance use of AI learning tools is not only a matter of technology acceptance at the tool level, but also reflects how learners connect their task competence with external learning support resources.

### 4.2 Physical activity self-efficacy as a domain-specific antecedent in TAM

One theoretical contribution of this study lies in clarifying why physical activity self-efficacy should be understood as a domain-specific psychological antecedent, rather than as a generic external variable or a direct substitute for technology self-efficacy, in explaining the continuance use of AI learning tools among physical education students. Previous technology acceptance research has often focused external variables on factors such as system characteristics, organizational support, or information quality. However, the present study shows that learners’ own belief foundations can also serve as an important source influencing the sustained adoption of technology (Rosli and Saleh, 2023; Alhwaiti, 2023). For physical education students, both learning and training are highly dependent on persistence, feedback regulation, task adjustment, and the ability to cope with performance-related challenges. Therefore, physical activity self-efficacy is related not only to students’ participation in training, but also to their readiness to use external support resources when facing learning and training difficulties. In this sense, AI learning tools may be perceived by students as feedback-seeking and problem-solving resources rather than as purely technological objects; however, this perception should be distinguished from objective evidence that such tools can provide accurate physiological monitoring or expert-level motor-skill feedback.

More specifically, physical activity self-efficacy significantly and positively predicted both perceived ease of use and perceived usefulness, with a stronger predictive effect on perceived ease of use. This suggests that, in the context of physical education, students tend to first form judgments about the controllability and manageability of a tool, and only then gradually develop evaluations of its practical value (Chen et al., 2025). At the same time, related studies have also indicated that self-efficacy can further shape individuals’ cognitive evaluations of a technology’s usefulness by influencing their sense of control over the technology (Kim and Kim, 2024). This finding not only supports the classical TAM logic that external variables enter the model through cognitive evaluations, but is also consistent with the path relationship in which perceived ease of use further influences perceived usefulness (Davis, 1989; Venkatesh and Davis, 2000; Chen et al., 2025). Theoretically, this finding extends the explanatory boundary of physical activity self-efficacy from exercise persistence and training participation to the sustained adoption of AI-supported learning tools. It suggests that, in physical education, students’ confidence in managing physical activity challenges may shape their perceived control over AI learning tools and their evaluations of whether such tools can support feedback seeking, learning reflection, training-related planning, and learning efficiency.

### 4.3 Mechanisms of multiple chained mediation pathways

Physical activity self-efficacy was associated with self-reported continuance use behavior of AI learning tools through five significant pathways, suggesting that this behavior may not be explained by a single mediating variable, but rather by a progressive mechanism jointly constituted by perceived ease of use, perceived usefulness, attitude toward use, and behavioral intention to use. Among these pathways, the indirect effect of path afh was relatively strong, highlighting the central role of perceived usefulness in linking physical activity self-efficacy to continuance use behavior (Alshammari and Babu, 2025). When physical education students possess higher levels of physical activity self-efficacy, they are more likely to regard AI learning tools as effective resources for supporting learning and training. This value judgment further strengthens their behavioral intention to use and ultimately translates into continuance use behavior (Chen et al., 2025).

At the same time, the significance of paths bdgh, bcegh, and bcfh indicates that perceived ease of use can not only directly influence attitude toward use, but can also promote subsequent behavioral transformation by enhancing perceived usefulness. In other words, in the context of physical education, students first form judgments about whether a tool is manageable and controllable, and then gradually develop evaluations of its actual value (Abbas et al., 2026). Attitude toward use and behavioral intention to use continued to play mediating roles across multiple pathways, indicating that continuance use behavior is not an automatic product of cognitive evaluation, but rather the result of a gradual progression from cognition to attitude and then to intention (Vashistha, 2025). Overall, these chained pathways support a continuance process with a clear temporal ordering: physical activity self-efficacy, as an antecedent psychological resource, was first associated with learners’ cognitive evaluations of AI tools and was then indirectly linked to self-reported continuance use behavior through attitude and intention. Although this sequence is consistent with the established TAM structure, the present findings add context-specific evidence by showing how a physical-domain efficacy belief may enter this process in AI-supported physical education.

Although the proposed model contains multiple chained mediation pathways, these pathways were retained for theoretical rather than statistical reasons. The model follows the sequential logic of TAM, in which perceived ease of use and perceived usefulness represent cognitive evaluations, attitude toward use reflects affective appraisal, and behavioral intention functions as the proximal antecedent of continuance use behavior. In this sense, the model complexity reflects the theoretical process through which physical activity self-efficacy is gradually translated into technology-related behavior, rather than an attempt to maximize statistically significant paths. Nevertheless, the relatively small indirect effects indicate that physical activity self-efficacy should be understood as one contributing factor rather than a dominant determinant of continuance use behavior. The fact that all hypothesized indirect paths were significant should therefore be interpreted as support for the theoretical consistency of the TAM-based process, rather than as evidence that the model provides a comprehensive explanation of continuance use behavior.

### 4.4 Theoretical and practical implications

The findings of this study have theoretical and practical implications for the application of AI learning tools in physical education contexts, although the practical implications should be interpreted in light of the modest indirect effects and the specific sample context. First, this study refines the application of TAM in physical education by showing how a physical-domain efficacy belief can be linked to technology continuance when AI learning tools are embedded in training feedback, skill learning, and physical education tasks. Therefore, the theoretical contribution is not limited to applying TAM to a new educational context, but lies in specifying how a physical education-specific action belief may enter the technology acceptance process. Unlike traditional studies that primarily focus on technological characteristics, the present study highlights the role of learners’ self-efficacy in the acceptance of AI learning tools. Specifically, physical activity self-efficacy among physical education students influenced their perceived ease of use and perceived usefulness of AI tools, and these cognitive factors, in turn, affected attitude toward use, behavioral intention to use, and continuance use behavior. Therefore, the value of the present model lies not in adding another predictor to TAM, but in specifying a context-sensitive pathway through which physical education students’ action beliefs are linked to sustained engagement with AI learning tools via perceived tool controllability and learning value.

From a practical perspective, the findings suggest that the design and promotion of AI learning tools may benefit from considering not only technological functions, but also students’ physical activity self-efficacy and learning confidence. For physical education students, AI tools should be regarded primarily as supplementary learning resources rather than substitutes for coaches, physiological monitoring, or professional training supervision. Therefore, when promoting AI tools, educators may consider students’ confidence and self-efficacy as supportive rather than decisive factors, while recognizing that these factors are likely to work together with tool quality, task fit, teacher support, and learning environment. For example, for students with low self-efficacy, barriers to use may be reduced through preliminary training, demonstration-based instruction, and task support, thereby strengthening their acceptance of such tools. At the same time, the design of AI learning tools should be aligned with the characteristics of physical education by improving interactivity, simplicity, and task fit, so as to enhance students’ behavioral intention to use and continuance use behavior. Carefully integrating AI tools with actual training tasks, under teacher or coach guidance where appropriate, may further strengthen students’ perceptions of their value and support their self-reported willingness and tendency to use such tools over time, although such practical applications should be viewed as incremental supports rather than stand-alone solutions.

## 5 Limitations

First, although the three-wave longitudinal design enhanced the explanatory power regarding the temporal ordering of variables, all variables, including continuance use behavior, were derived from self-reported measures. Therefore, the influence of subjective reporting bias, social desirability bias, recall bias, and shared method variance cannot be entirely ruled out. In particular, the continuance use behavior measured in this study reflects students’ self-reported frequency, duration, and functional scope of AI tool use rather than objectively recorded usage behavior. Future research should incorporate multiple data sources, such as behavioral logs, platform usage data, teacher evaluations, or learning analytics records, to further improve the robustness of the findings. Moreover, this study did not collect physiological monitoring data, biomechanical data, objective performance indicators, or real-time motor-feedback records. Therefore, the findings should be interpreted as evidence regarding students’ acceptance and self-reported continuance use of AI learning tools, rather than evidence that AI tools directly improve motor performance or provide scientifically validated individualized training feedback.

Second, the sample was drawn exclusively from Chinese physical education students, and most participants were already frequent users of AI learning tools, which limits the generalizability of the findings. Physical education students are characterized by practice-oriented learning, frequent training tasks, and strong reliance on feedback, and these characteristics may differ from those of students in more theory-oriented disciplines. Therefore, the findings should not be directly generalized to students from other academic disciplines, educational stages, age groups, cultural contexts, or populations with limited prior experience using AI learning tools. Future studies should replicate this model among students from different majors, non-physical education populations, cross-cultural samples, and students with varying levels of prior AI tool use to examine the broader applicability of the proposed mechanism.

Finally, this study incorporated physical activity self-efficacy into the TAM framework as a key antecedent variable and focused on the pathways through which it influences continuance use behavior via perceived ease of use, perceived usefulness, attitude toward use, and behavioral intention to use. Although this approach helped highlight the core mechanism, it did not include potential boundary conditions such as AI tool type, task complexity, teacher support, or differences in learning environments. Future research may introduce moderating variables or adopt multi-group comparison designs to more precisely identify the conditions under which continuance use behavior of AI learning tools is formed. In addition, although all chained mediation pathways were statistically significant, the indirect effects were relatively small. This suggests that the proposed mechanism should be interpreted as a modest explanatory pathway rather than a comprehensive or dominant explanation of continuance use behavior. Future research may compare the present model with more parsimonious alternative models or incorporate additional contextual factors to further clarify the relative explanatory power of different pathways. In addition, the term “AI learning tools” covered heterogeneous tools, including general AI question-answering systems, learning-planning applications, assignment-feedback tools, wearable devices, and motion-feedback devices. Because the present study did not distinguish the technical capabilities or accuracy levels of these tools, future research should compare different types of AI tools and examine whether their recommendations are valid, personalized, and safe in physical education settings.

## 6 Conclusion

Physical activity self-efficacy may serve as a statistically significant but relatively modest antecedent factor associated with self-reported continuance use behavior of AI learning tools among physical education students. Physical activity self-efficacy not only positively predicted perceived ease of use and perceived usefulness, but was also associated with self-reported continuance use behavior through multiple chained pathways involving perceived ease of use, perceived usefulness, attitude toward use, and behavioral intention to use. All five hypothesized pathways were supported, suggesting that the continuance use of AI learning tools may not be explained by a single factor alone, but may involve a time-separated process from cognitive evaluation to attitude formation and then to behavioral intention.

Overall, the present study shows that physical activity self-efficacy can be theoretically linked to the continuance use of AI learning tools when it is understood as a physical education-specific action belief that shapes learners’ perceptions of tool controllability, usefulness, and subsequent behavioral commitment. For physical education practice, promoting the long-term use of AI learning tools may require not only optimizing tool ease of use and perceived usefulness, but also considering learners’ action beliefs, sense of task competence, teacher guidance, and the limitations of AI-generated information.

## Data Availability

The datasets presented in this study can be found in online repositories. The names of the repository/repositories and accession number(s) can be found below: The dataset generated and/or analyzed during the current study has been publicly released and is freely available on Figshare at: https://doi.org/10.6084/m9.figshare.31908673.
